# Coupling Effect of Porosity and Cell Size on the Deformation Behavior of Al Alloy Foam under Quasi-Static Compression

**DOI:** 10.3390/ma12060951

**Published:** 2019-03-21

**Authors:** Donghui Yang, Hui Wang, Sensen Guo, Jianqing Chen, Yongmin Xu, Dong Lei, Jiapeng Sun, Lei Wang, Jinghua Jiang, Aibin Ma

**Affiliations:** 1 College of Mechanics and Materials, Hohai University, Nanjing 211100, China; yang_donghui76@hotmail.com (D.Y.); muzhiji@outlook.com (S.G.); chenjq@hhu.edu.cn (J.C.); 15850686895@163.com (Y.X.); leidong@hhu.edu.cn (D.L.); 20140070@hhu.edu.cn (J.S.); wangl@hhu.edu.cn (L.W.); jinghua-jiang@hhu.edu.cn (J.J.); aibin-ma@hhu.edu.cn (A.M.); 2 State Key Laboratory for Advanced Metals and Materials, University of Science and Technology Beijing, Beijing 100083, China; 3 Suqian Research Institute of Hohai University, Suqian 223814, China

**Keywords:** al alloy foam, compression, hierarchical pore structure, digital image correlation

## Abstract

Closed-cell AlCu5Mn alloy foam with porosity range of ~45–90% were fabricated by the melt-foaming route. The pore structure of the fabricated Al alloy foam was analyzed and the coupling effect of porosity and cell size on the quasi-static compression behavior of the foam was investigated. The results show that the cell size of the foam decreases with the porosity decline from the view of the contribution rate to the porosity and the hierarchical pore structure characteristics becomes obvious when the foam porosity is low; the compression stress–strain curves of the foams with high porosity (>74%) are serrated due to the large cell size being easy to deform and more strain needed to let the stress recover. Meanwhile, the compression curve of the foams with low porosity (<74%) are smooth without serration, which is attributed to the hierarchical pore structure and less strain needed to let the stress recovery.

## 1. Introduction

Al alloy foam is regarded as an attractive material for its ultra-light weight, high specific strength and good energy absorption capacity during the compression [1,2,3,4,5]. Al alloy foam can be produced by a variety of methods, e.g., pressure infiltration method [6,7], lost casting method [8], space holder technique [9], powder sintering and dissolution route [10] for preparing the Al foam with open pore structure, gravity casting technique [11,12,13] for making metal matrix syntactic foams, powder metallurgical route [14,15,16], gas injection method [17,18] and melt-foaming method [19,20,21,22] for fabricating Al foam with a close-cell. Among them, the melt-foaming (MF) method (Alporas route) by using titanium hydride as the blowing agent is an effective approach to fabricate close-cell Al alloy foam because of its relatively low cost.

The compression deformation behavior of Al alloy foams is strongly affected by both the matrix property and the foam pore structure [22]. So far, extensive researches have been reported on the compression deformation property of the close-cell Al alloy foams with high porosity (>85%) and large cell size (2–6 mm) [21,22,23,24,25,26], and three deformation modes have been proposed [27], which are (i) bending and formation of a plastic hinge at the cell edge and cell membrane; (ii) ductile tearing and fracture caused by the tension and shearing; (iii) stretching and buckling of the cell membrane. However, the deformation characteristics of close-cell Al alloy foam with low porosity and small cell size has not been well investigated.

In this work, the AlCu5Mn alloy foams with wide range porosity (~45%–90%) were fabricated first by the MF route. Then, the pore structure (porosity, pore cell size distribution and its contribution rate to the porosity) was analyzed, the compression behavior of the foam was examined and the DIC (digital image correlation) was utilized to examine the full field deformation characteristics of the foam compressed to the yield point. Finally, the coupling effect of porosity and cell size on the compression behavior of Al alloy foam was discussed.

## 2. Experiments

### 2.1. Close-Cell Al Alloy Foam Fabrication

AlCu5Mn alloy with composition of 4.5–5.3 wt.% Cu, 0.6–1.0 wt.% Mn and 0.15–0.35 wt.% Ti, which possesses good mechanical property [28,29], was used as the starting material. Ca particle (purity > 99.0%, 2 mm) was selected as the thickening agent and the as-received titanium hydride powder (purity > 99.2 wt.%, 40 μm) was chosen as the blowing agent. The MF route was applied to fabricate the Al alloy foam and the specific preparation process has been reported in Reference [30]. Briefly, (1) the Al alloy was first melted and thickened by adding 2.0 wt.% Ca particle. (2) Then, titanium hydride was added and distributed in the thickened Al alloy melt by the impellor with revolution speed of 1000 rpm, leading to the melt being foamed gradually. This time interval is defined as the stirring foaming stage, in which duration is defined as the stirring foaming time. (3) The impellor was pulled out and the foamed melt was kept at a proper temperature to keep titanium hydride decomposing, which makes the bubbles in the melt grow and form a cellular structure. This time interval is defined as the holding foaming stage, in which duration is defined as the holding foaming time. (4) Finally, the foam sample can be obtained after the foamed melt is solidified. The Al alloy melt-foaming process is determined by the titanium hydride decomposition kinetics, and the pore structures of the final foam can be controlled by adjusting the stirring foaming time, the holding foaming time, impellor stirring speed and the moment at which the foamed melt is initiated to be solidified [30,31].

### 2.2. Pore Structure Characterization

The porosity (*Pr*), the pore fraction of a foam specimen, was calculated from its mass (*M*) and volume (*V*) by using the equation of *Pr* (%) = (*V* − *M*/*ρ*_s_)/*V* × 100%, where ρs is the matrix density (i.e., 2.78 g/cm^3^). The plane pore cell size of a fabricated foam was calculated by analyzing its cross-section stereo micrographs with the aid of image analysis software. The plane pore cell size was used to reflect the foam bulk pore cell size (*D*) and the specific treatment has been presented in Reference [32]. Briefly, the macro-pore-structure of the foams was observed by KH-7700 Stereo-type microscope (QUESTAR China Limited, Shanghai, China) first, and because the reflecting light ability of the cell edge is stronger than that of the inner surface of the pore, the obtained stereo micrograph of the foams can be analyzed by the Image Pro Plus software to acquire the plane pore cell size.

### 2.3. Quasi-Static Compression Experiment

The cubic specimens with a size of 30 mm × 30 mm × 30 mm were machined by electro-discharging machine and the specimen size is 10 times larger than the average cell size, which can avoid the size effect on its mechanical property. The quasi-static compression was carried out at room temperature on the Electronic Universal Material Testing Machine (Instron 3360 Series Testing Machine, Norwood, MA, USA) at the rate of 1 mm/min. During the compression tests, the high precision CCD camera with the M0814-MP’s megapixel lens of Japan Computer was used to record a surface of the specimen. The resolution of each image was 1624 × 1224 pixel^2^ and in this study, one pixel represents 0.04 mm. The strain resolution of this DIC system is 0.01%.

## 3. Results and Discussion

### 3.1. Pore Structure of AlCu5Mn Alloy Foam

**Figure 1:**
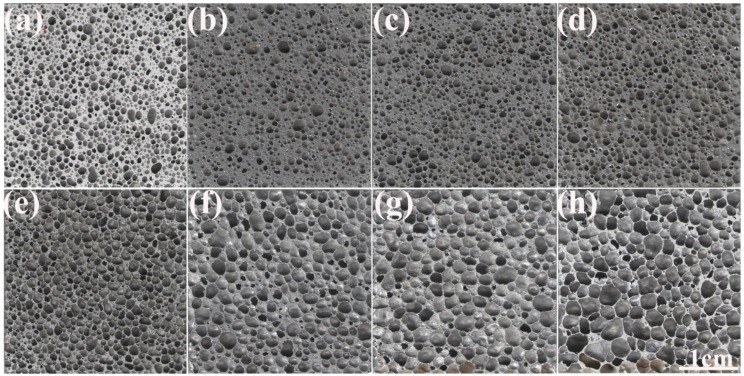
Section images of AlCu5Mn alloy foam with porosities of (**a**) 58.5%, (**b**) 61.2%, (**c**) 68.0%, (**d**) 72.9%, (**e**) 76.5%, (**f**) 80.9%, (**g**) 83.2% and (**h**) 87.5%.

Figure 1 shows the section images of AlCu5Mn alloy foams with porosity from 58.5% to 87.5%, which demonstrates that all the samples have homogeneous pore structure, and the cell shapes evolve into polygons with foam porosity increase. Additionally, according to Figure 1, it seems that there is a tendency for the cell size to become larger with porosity increase.

**Figure 2:**
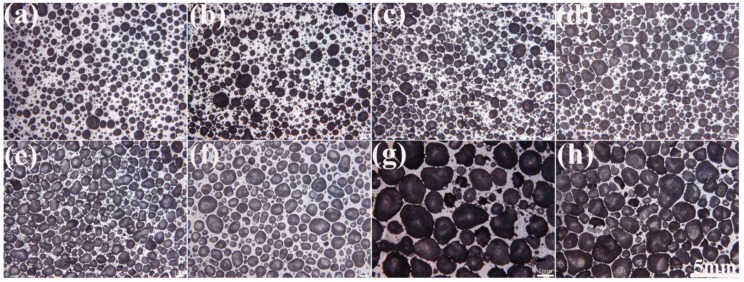
Stereo micrographs of AlCu5Mn alloy foam with porosities of (**a**) 58.5%, (**b**) 61.2%, (**c**) 68.0%, (**d**) 72.9%, (**e**) 76.5%, (**f**) 80.9%, (**g**) 83.2% and (**h**) 87.5%.

**Figure 3:**
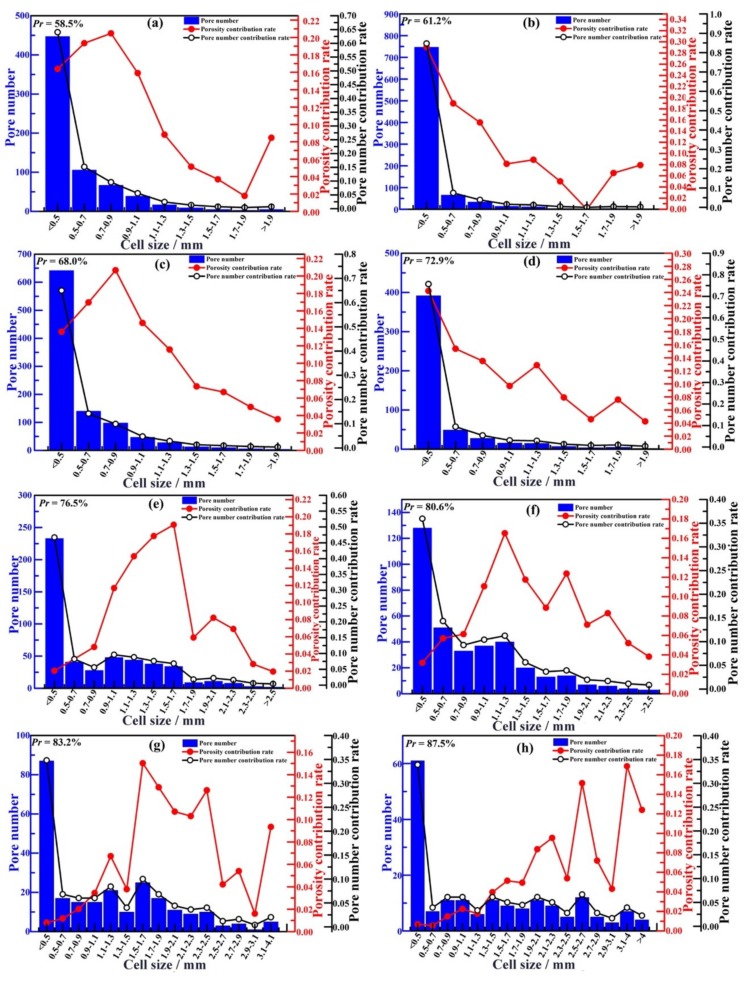
Pore cell size distribution and the corresponding contribution rate to the porosity of the AlCu5Mn alloy foam with porosities of (**a**) 58.5%, (**b**) 61.2%, (**c**) 68.0%, (**d**) 72.9%, (**e**) 76.5%, (**f**) 80.9%, (**g**) 83.2% and (**h**) 87.5%.

Figure 2 demonstrates the corresponding stereo micrographs of the AlCu5Mn alloy foams shown in Figure 1. After image analysis, the pore cell size distribution, the pore number contribution rate and the contribution rates to the porosity by different pore cell size are exhibited in Figure 3.

According to Figure 3, the pore number contribution rate by the pores with *D* < 1.1 mm accounts for more than 0.5 for all the foams. However, the contribution rates to the porosity by the pores with *D* < 1.1 mm for the foam with *Pr* < 73% and *Pr* > 75% are different, which are higher than 0.63 and lower than 0.075, respectively. Specifically, for the foam with *Pr* < 73%, the pores with *D* < 1.1 mm contribution rates to the pore number are all higher than 0.93, and their corresponding contribution rates to the porosity are 0.72, 0.72, 0.66 and 0.63 for the foams with porosities of 58.5%, 61.2%, 68.0% and 72.9%, respectively. Meanwhile, for the foams with *Pr* > 75%, although the pores with *D* < 1.1 mm contribution rates to the pore number are all higher than 0.5, their corresponding contribution rates to the porosity are only 0.020, 0.026, 0.074 and 0.050 for the foams with porosities of 76.5%, 80.9%, 83.2% and 87.5%, respectively.

**Figure 4:**
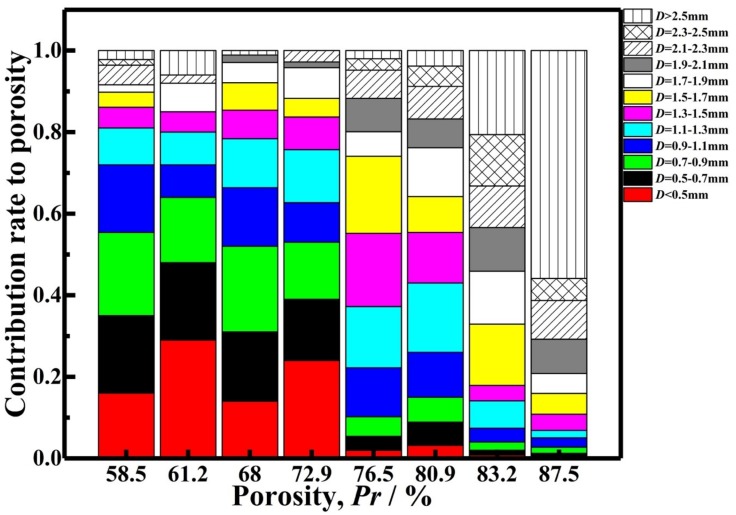
Contribution rates to the porosity by the pores with different cell size (*D*) for AlCu5Mn alloy foams with porosities of 58.5%, 61.2%, 68.0%, 72.9%, 76.5%, 80.9%, 83.2% and 87.5%.

Furthermore, according to data in Figure 3, the contribution rate to the porosity by the pores with different cell size for AlCu5Mn alloy foams with porosity of 58.5–87.5% is summarized and displayed in Figure 4, which indicates there is a tendency that with the foam porosity increasing, the contribution rate to the porosity by the larger pores increases. Therefore, from the view of contribution rate to the porosity, it can be safely assumed that the cell size of the AlCu5Mn alloy foam fabricated by the MF method has the tendency to decrease with the porosity decline or vice versa.

### 3.2. Compression Behavior of AlCu5Mn Alloy Foam

**Figure 5:**
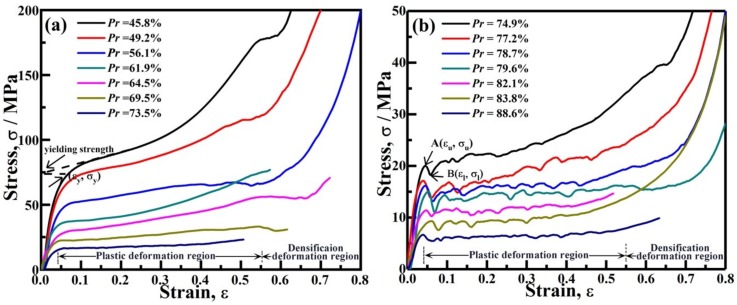
Compression stress-strain curves of AlCu5Mn alloy foams with porosity of (**a**) 45.8%–73.5% and (**b**) 74.9%–88.6%. Some stress–strain curves stopped around the onset densification region due to the sample collapse.

Figure 5 is the compression engineering stress (σ)–strain (ε) curve of AlCu5Mn alloy foam with porosity of 45.8%–88.6%, which demonstrates that the compression stress-strain curves of the foams consist of three regions: (i) an initial approximately linear deformation region, where the relation between stress and strain is approximately linear, (ii) plastic deformation region, where the stress increases slowly or even remains constant with the strain increasing and (iii), densification deformation region, where stress increases rapidly with little increase of strain due to most cell having been crushed and the cell walls having touched each other.

It is worth noting that the compression stress-strain curves of the foams with low porosity (e.g., *Pr* < 74%) is different from those of the foams with high porosity (e.g., *Pr* > 74%). The foams with *Pr* < 74% as shown in detail in Figure 5a; their compression stress-strain curves increase sufficiently smoothly; while for the foams with *Pr* > 74% as shown in Figure 5b, a pair of local peak stress A (σ_u_) and minimum stress B (σ_l_) appears, and the compression stress-strain curves during the plastic deformation stage are serrated. Therefore, the yielding strength (σ_y_) of the Al alloy foam with porosity lower than 74% is defined by extrapolating the plateau regime to ε = 0, as shown in Figure 5a. For the Al alloy foam with porosity higher than 74%, the σ_u_ is used to reflect the foam yielding strength [2]. Accordingly, Figure 5 indicates that the yield strength of the foam decreases with its porosity increase, where the yield strength decreases from 82.7 MPa to 6 MPa with the porosity increasing from 45.8% to 88.6%, and the yield strain varies in the range of 0.04–0.06.

### 3.3. Coupling Effect of Porosity and Cell Size on Compression Property of AlCu5Mn Alloy Foam

A foam is considered to be brittle when its compression stress-strain curve possesses the local peak/minimum stress and is serrated during the plastic deformation stage. Therefore, AlCu5Mn foam with porosity higher than 74% is supposed to be “brittle”. However, as shown in Figure 5b, when the foam porosity lower than 74%, there is no such local peak/minimum stress and the compression stress-strain curves are smooth, which indicates that the foam is not “brittle” anymore. 

**Figure 6:**
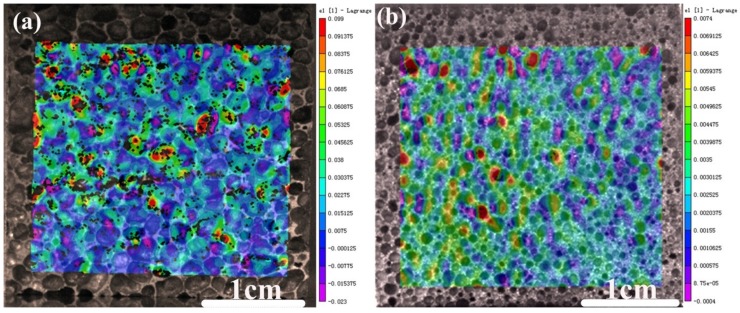
Principle strain distributions of the AlCu5Mn alloy foams with porosities of (**a**) 83.2% and (**b**) 61.9% at the yield strains of 0.045 and 0.06, respectively.

Figure 6 shows the principle strain distributions of the AlCu5Mn alloy foams with porosities of 61.9% and 83.2% compressed to the yield points with strains of 0.045 and 0.06, respectively. As shown in Figure 6a, for the foam with porosity of 83.2% compressed to its yield point (ε_u_ = 0.06), the principle strain of some pores can reach ~0.1, indicating that those pores should have been crushed already. Meanwhile, as shown in Figure 6b, for the foam with porosity of 61.9% compressed to its yield point (ε_y_ = 0.045), the principle strain is much lower and maximum principle strain is only ~0.007. The above results imply that the coupling effect of porosity and cell size on the compression behavior of the AlCu5Mn foam should be considered.

**Figure 7:**
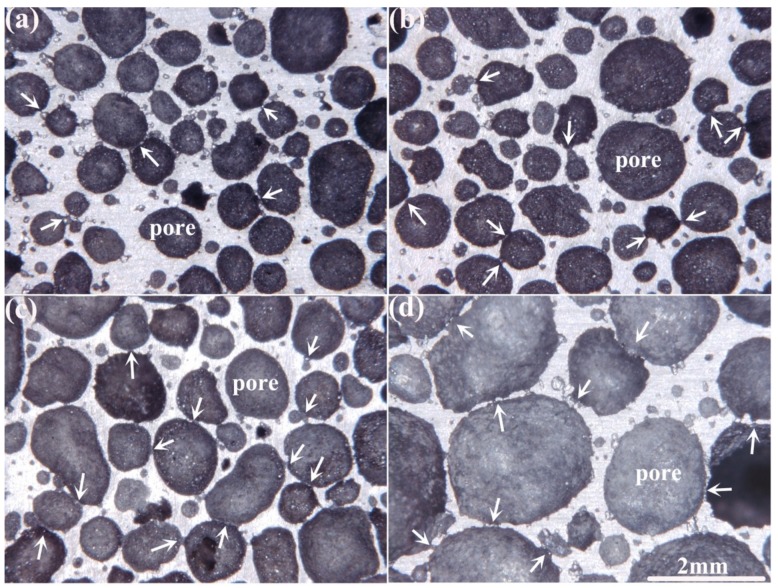
Stereo micrographs of AlCu5Mn alloy foams with porosities of (**a**) 58.5%, (**b**) 68.0%, (**c**) 76.5% and (**d**) 83.2%.

As mentioned in Section 3.1, the pore cell size decreases with porosity decline. Furthermore, as shown in Figure 7, the high magnification stereo micrographs of AlCu5Mn alloy foams with porosities of 58.5%, 68.0%, 76.5% and 83.2%, the foams with low porosity possess the hierarchical pore structure. For the foams with porosities of 58.5% and 68.5%, the contribution rates to the porosity by the pore with cell size smaller than 1.1 mm is 0.72 and 0.66, respectively (data from Figure 3), and it can be seen from Figure 7a and b that these two foams have the hierarchical pore structure, where the cell size difference among the pores smaller than 1 mm can be more than 2 times. For the foams with porosities of 76.5% and 83.2%, the contribution rates to the porosity by the pores larger than 1.1 mm is higher than 0.75 (data from Figure 3). Furthermore, Figure 7c,d shows the hierarchical pore structure characteristics, where the cell size difference among the pores with size larger than 1 mm is less than 2 times, is not obvious.

Additionally, as shown by the arrows in Figure 7, the broken cell walls can be observed in all the foams. Obviously, for the foam with high porosity (i.e., *Pr* = 83.2% in Figure 7), most broken cell walls exist between the pores with cell size larger than ~2 mm, while for the foams with low porosity (i.e., *Pr* = 58.5% and 68.0% in Figure 7), the broken cell walls exist between the pores with size smaller than 1 mm.

**Figure 8:**
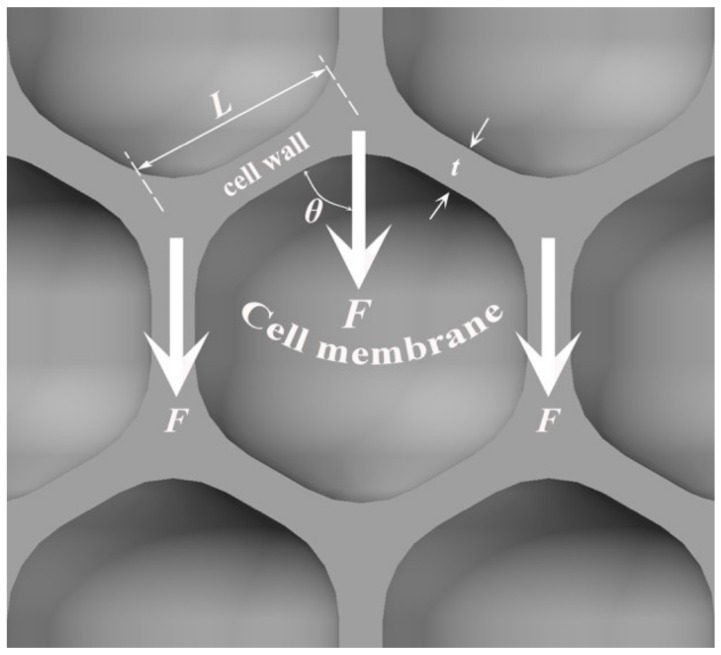
Compression model of metallic foam.

As shown in Figure 8, when the compression force *F* is applied on the foam, the force direction can be parallel with, or has an angle *θ*, with a cell wall. Then, the strength of a foam is determined by the cell wall strength and the moment upon them. The moment (*M*) upon the cell wall is expressed as:

*M* = *F*⋅sin *θ* × *L* (1)

where *F* is the compressive force on the cell wall, *L* is the cell wall length and the *θ* is the angle between the *F* direction and the cell wall.

When the compressive stress reaches a foam’s yield strength, a band of cell walls and cell membrane will buckle/fracture/crumple [27] and the stress will be released temporarily, which will cause the stress to drop suddenly. After the broken cell walls or the cell membranes are compacted and the proximate cells of compacted layer contacts each other, the stress will recover. The compaction distance for the pore with large cell size is longer, which implies that more strain is needed to make the stress recover for the foam with high porosity and thus, a stress sudden drop can be observed in the compression stress-strain curve, as shown in Figure 5b.

When the foam porosity declines, the pore cell size decreases, the corresponding metal matrix fraction, the average thickness of the cell wall (*t* shown in Figure 8) and the cell membrane thickness will increase while the length of the cell walls (*L* shown in Figure 8) decrease. Therefore, for the foam with low porosity, a higher compressive force *F* is needed to make a short and thick cell wall buckle/fracture. According to Equation (1), for a given *θ* and *t*, the shorter the cell wall (*L*), the moment applied on the cell wall is smaller. Additionally, foams with low porosity have the hierarchal pore structure and the small cell size (e.g., *D* < 1 mm). Although the cell walls of the pores with relatively large size/thin cell walls/broken cell walls prefer to buckle or fracture during the yielding process, the cell walls of the nearby relatively small pores/thick cell walls can bear the load without buckling or fracturing. Consequently, the yield strength of AlCu5Mn foam with lower porosity is higher, as shown in Figure 5. Furthermore, the strain needed for stress recovery is smaller than those foams with high porosity. Thus, the sudden drop in stress does not appear in the compression stress–strain curve of the AlCu5Mn alloy foam with low porosity, as shown in Figure 5a.

## 4. Conclusions

Close-cell AlCu5Mn foam with porosity range of 45–89% were fabricated successfully by the melt-foaming route. The pores with small cell size (*D* < 1.1 mm) contribution rate to the pore number accounts for over 0.5 for all the fabricated foams. For the foam with porosity lower than 73%, its porosity is mainly contributed by the pores with small cell size (*D* < 1.1 mm) and the hierarchical pore structure can appear. However, for the foam with porosity higher than 75%, the contribution rate to the porosity by the pores with small cell size (*D* < 1.1 mm) is lower than 0.063 and the hierarchical pore structure characteristics are not obvious. Because the contribution rate to the porosity by the pores with large cell size increases with porosity increasing, it is reasonable to conclude that the cell size of the foam decreases with the porosity decline.

Three regions in the quasi-static compression stress-strain curve of AlCu5Mn alloy foam exist: approximately linear deformation stage, plastic deformation region and densification region. The yield strength of the AlCu5Mn alloy foam decreases with porosity decrease and the yield strain varies in the range of 0.04–0.06. 

During the compression process, the pores with large cell size are easy to collapse, leading to the stress being released. Thus, more strain is needed to make the stress recover for the foams with high porosity. Consequently, a pair of local peak and minimum stresses appear when the foam with high porosity (*Pr* > 74%) is compressed to the yield point and stress-strain curve is serrate during the plastic deformation region. In the case of the foam with low porosity (*Pr* < 74%), the small cell size and the hierarchical pore structure make the corresponding compression stress-strain curve smooth because the strain needed to for stress recovery is smaller.
